# The deadly triple M (mistrust, misinformation, and missed opportunities): understanding Romania’s COVID-19 vaccination campaign and its lasting impact on public health

**DOI:** 10.3389/fpubh.2025.1631799

**Published:** 2025-09-17

**Authors:** Stefan Dascalu, Catalin Valentin Raiu, Emilian Olteanu, Adrian Vasile Comanici, Mihaela Maria Comanici, Tudor P. Toma, Bogdan Ionut Robu, Raul Mihailov, Laura Mina-Raiu, Gindrovel Gheorghe Dumitra, Doina Azoicai, Emilian Damian Popovici, Cristian Apetrei

**Affiliations:** ^1^Department of Biology, University of Oxford, Peter Medawar Building for Pathogen Research, Oxford, United Kingdom; ^2^Department of Public Administration, Faculty of Business and Administration, University of Bucharest, Bucharest, Romania; ^3^Department of Pathology, British Columbia Cancer Agency, Vancouver, BC, Canada; ^4^Department of Pathology and Laboratory Medicine, University of British Columbia, Vancouver, BC, Canada; ^5^Department of Endocrinology, “Titu Maiorescu” University of Medicine and Pharmacy, Bucharest, Romania; ^6^Department of Endocrinology, C.F.2 Clinical Hospital, Bucharest, Romania; ^7^Department of Immunology, “Titu Maiorescu” University of Medicine and Pharmacy, Bucharest, Romania; ^8^Lewisham and Greenwich NHS Trust, London, United Kingdom; ^9^King’s College, London, United Kingdom; ^10^Great Western Hospitals NHS Foundation Trust, Swindon, United Kingdom; ^11^Faculty of Medicine and Pharmacy, Dunarea de Jos University, Galați, Romania; ^12^Clinical Emergency Hospital “Sf. Ap. Andrei”, Galați, Romania; ^13^Bucharest University of Economic Studies, Bucharest, Romania; ^14^Family Medicine Department, University of Medicine and Pharmacy of Craiova, Craiova, Romania; ^15^Department of Epidemiology, School of Medicine, Grigore T. Popa University of Medicine and Pharmacy, Iași, Romania; ^16^Department of Epidemiology, School of Medicine, Victor Babeş University of Medicine and Pharmacy, Timişoara, Romania; ^17^Regional Centre of Public Health, Timişoara, Romania; ^18^Division of Infectious Diseases, School of Medicine, University of Pittsburgh, Pittsburgh, PA, United States; ^19^Department of Infectious Diseases and Immunology, Graduate School of Public Health, University of Pittsburgh, Pittsburgh, PA, United States

**Keywords:** public health, Romania, misinformation, COVID-19 vaccination, vaccine hesitancy

## Abstract

Romania’s COVID-19 vaccination campaign presents a compelling case study on the intersection of public health policy, societal dynamics, and political influences in pandemic response. Despite an initially promising rollout, Romania ultimately achieved one of the lowest vaccination rates in the European Union, with severe consequences during the subsequent pandemic waves. This review examines the key factors contributing to the campaign’s shortcomings, including pre-existing vaccine hesitancy, widespread misinformation, inadequate governmental communication strategies, and the politicisation of public health efforts. We explore the deep-seated mistrust in governmental institutions, exacerbated by restrictive measures implemented without adequate public engagement, as well as the influential role of religious communities and the rise of populist political forces that actively opposed vaccination efforts. Additionally, we discuss the impact of media sensationalism, conspiracy theories, and the failure to regulate anti-vaccine rhetoric within the medical profession. While logistical and infrastructural challenges were largely addressed, the inability to effectively engage key societal stakeholders led to lagging of vaccine uptake. The consequences of this failure extended beyond COVID-19, contributing to a severe measles outbreak in 2023, which underscored the long-term deleterious effects of vaccine hesitancy. Drawing from Romania’s experience, we highlight critical lessons for future public health campaigns, emphasising the need for trust-building initiatives, targeted misinformation countermeasures, stronger community engagement, and enhanced collaboration with religious and cultural institutions. By addressing these challenges, countries worldwide can strengthen their public health frameworks and improve the resilience of their immunisation programmes in the face of future crises.

## Highlights

We identify and analyze a “deadly triple M” (mistrust, misinformation, and missed opportunities) that collectively undermined vaccine uptake and left lasting impacts on public health. The analysis is organized across four distinctive time periods:

- Pre-COVID pandemic, when inadequate public health measures,pre-existing conceptions, prior national outbreaks (e.g. measles), and entrenched anti-vaccine narratives shaped public attitudes.
- Early COVID pandemic (before the vaccination campaign), when restrictive measures, political instability, and inconsistent communication eroded public trust.
- Mid-late COVID (during the vaccination campaign), when politicization, inadequate engagement with community stakeholders (including religious communities), and the influence of anti-vaccine voices stalled uptake.
- Post-pandemic, when low MMR vaccine coverage contributed to a severe measles outbreak in 2023, demonstrating the long-term spillover effects of pandemic-era misinformation. Drawing on these findings, the paper outlines policy implications and offers eight targeted recommendations aimed at rebuilding trust, improving stakeholder engagement, countering misinformation, and strengthening Romania’s capacity to respond to future public health crises. These lessons hold broad international relevance for countries facing similar challenges in vaccine acceptance and public health communication.

## Introduction

National COVID-19 vaccination campaigns began throughout the world as the vaccines became available by the end of 2020, the first year of the COVID-19 pandemic (1). These vaccination campaigns had variable degrees of success in terms of their implementation and, ultimately, in terms of the overall immunisation coverages achieved (2). Romania suffered one of the highest per capita COVID-19 death toll worldwide during the Delta wave of the pandemic (3), and a substantial level of COVID-19 mortality during the Omicron wave, in spite of the relative decrease in disease severity associated with this latter variant (4). Paradoxically, during both Delta and Omicron waves, effective COVID-19 vaccines were already widely available and the national vaccination programme was well under way (4, 5).

Several unique demographic and socio-economic characteristics made Romania stand out with regards to the implications for public health management, especially given its very large and widespread diaspora (6, 7). This population of Romanian expatriates dramatically impacted the initial spread of SARS-CoV-2, the virus that causes COVID-19, during the early days of the pandemic, when citizens returned *en masse* to Romania from multiple destinations, some of them highly affected by the virus (8). As such, multiple founder events triggered the Romanian epidemic (9, 10), posing significant concerns to the public health authorities. These multiple epidemic occurrences generated hardship for Romania’s health system, already severely underfinanced, with a health spending allocation of 5.7% of the GDP in 2019 (11), and facing the highest rates of treatable and preventable mortality within the European Union (6).

The COVID-19-related pressures exerted on the healthcare system peaked successively during each of the first four waves of the pandemic, which were caused by the spread of different virus variants (12–14). These four waves yielded multiple differences, in terms of both virus characteristics and the degree of population immunity (5, 15–19). In 2020, during the first two waves, associated with the original SARS-CoV-2 variant, the overall levels of population immunity were very low (15, 16). In addition, the only means for prevention and control of COVID-19 infections at the time were through generic measures, such as social distancing, masking, and lockdowns (20, 21).

The national COVID-19 vaccination campaign began in Romania on the 27th of December 2020 and was coordinated by the Romanian National Committee on COVID-19 Vaccination (CNCAV) (5). As a result, the third wave caused by the Alpha variant of the virus (B.1.1.7), which occurred during the winter/spring of 2021, encountered both a certain level of natural immunity and a gradually rising vaccine-induced immunity (22). However, it was the fourth wave that hit Romania the hardest. At the time of the spread of the Delta variant towards the end of 2021, the Romanian population had achieved only little over 40% vaccination (23), following the hurdles associated with the vaccination campaign (5, 24).

Meanwhile, the fifth COVID-19 wave, primarily caused by the Omicron variant in early 2022, found a population which was mainly immunised naturally, and only partly through vaccination (25, 26). As a result, the fifth wave was relatively mild in terms of both its magnitude and its severity. In December 2022, CNCAV released an activity report entitled *“Rovaccination. Mission accomplished”* (27). However, at the time of the report’s publication, Romania was among the European countries with the lowest COVID-19 vaccination coverages (23).

In this paper, we present a critical narrative review structured around a chronological framework which examines key events and their implications for Romania’s COVID-19 vaccination campaign. We reviewed policy documents, peer-reviewed academic literature, and non-academic public sources (including press coverage) to capture public perceptions, narratives and policy/governmental positioning. These materials were critically analysed to assess the public health impact of significant pandemic events on vaccination uptake and societal perceptions of vaccination in Romania. Based on our analysis, we formulated targeted recommendations to address identified challenges and strengthen future public health responses.

### Pre-COVID-19: historical context and pre-existing vaccine narratives in Romania

Romania’s prepandemic profile did not place the country in a favorable position to manage the consequences of COVID-19. Indeed, the country was already facing major challenges with regards to the provisioning of hospitals, their adequate staffing, vaccine acceptance by the overall population, and appropriate funding of the health sector as a whole (6). With regard to the infectious diseases management, just a few years before the COVID-19 pandemic, between 2016 and 2019, Romania faced one of the worst European measles epidemics of the current century, with more than 17,000 cases and 59 deaths (28). Additionally, seeding events occurred in many other countries, both within Europe and beyond. The major cause of the devastating measles epidemic was the decline in measles, mumps, and rubella (MMR) vaccination coverage, which had maintained suboptimal levels for at least 6 years before the epidemic began (28). Unfortunately, the measles debacle was insufficient to prompt adequate measures of epidemic preparedness in terms of either prevention and control measures *per se*, or public awareness towards vaccine-preventable diseases (5, 6, 29). These failures had dramatic consequences during the COVID-19 pandemic a few years later, as both the healthcare system and Romanian society were underprepared for the crisis that would unfold.

An important catalyst of the 2016–2018 measles epidemic and, later on, a driver of the COVID-19 vaccination outcomes was the general public perception of vaccinations in Romania (28, 30). From the early 2000s, the anti-vaccine movement has slowly but firmly embedded itself in Romanian society, gaining support from average citizens, influencers, and even medical professionals. As such, Romania is a case in point for the devastating consequences of the “bad advice” [see ref. (31)]. As a result, the past two decades have seen not only a major increase in anti-vaccine propaganda, such as NGOs, books, websites, social media pages, but also the aggressive mediatization of anti-vaccine influencers, some of them with a medical degree, which further exacerbated the already negative trends in vaccine acceptance, especially in terms of childhood vaccination program (6, 28, 32). For measles, this culminated with the 2016–2018 epidemic, but other public health prevention and control efforts have also been hindered. In 2008, the national human papillomavirus (HPV) vaccination campaign achieved little over 2% coverage of the target population of 9- to 11-year-old girls (33). As the anti-vaccine phenomenon has been virtually unaddressed by the authorities, the efforts of healthcare professionals and other vaccine advocates, as well as the national vaccination campaigns carried out in Romania prior to the COVID-19 pandemic, have had limited effects on overall vaccine acceptance (28, 29, 34).

## The first year of the pandemic in Romania: towards a COVID-19 vaccine amid trust erosion and public health challenges

### Restrictive measures and erosion of public trust in Romanian authorities

The prepandemic hesitancy of the Romanian population towards vaccinations was further fueled by the severe restrictions and lockdowns implemented during the first year of the COVID-19 pandemic (6). These were not particular to Romania, as they were implemented worldwide with the goal of halting/slowing the spread of the epidemic. Specific to Romania is the sizable diaspora. Many Romanians returned from abroad, often from highly affected regions, driven by the fear of infection, the threat of being isolated in a foreign country, and the economic insecurity associated with lockdowns. However, the major threats to public health were controlled by implementing harsh quarantine rules for returning citizens and, later on, general lockdowns (6, 35). With this in mind, while the first lockdown and governmental restrictions on individual freedoms were understandably implemented on an emergency basis through military ordinances (6), these legislative circumstances did not change for the subsequent lockdown and restrictions, and a legal, more democratic framework was not created for their implementation.

This triggered a general sentiment of frustration and mistrust in national authorities, which was revealed by a survey carried out in May 2020, just after the first lockdown, when only 36% of respondents favorably viewed the Government as a trustful partner following the early COVID-19 crisis (36). Unfortunately, this impacted both public trust and compliance—feelings which were reflected in peoples’ attitudes towards the vaccine. In the same survey mentioned above, only 44% of Romanians declared that they would be willing to receive a vaccine, should a vaccine become available, with another 22% stating that they would be hesitant towards this prospect (36). As such, it is likely that the cumulative effect of the early pandemic responses was the exacerbation of mistrust in the government and public health authorities, especially given the parallels that were drawn to Romania’s authoritative communist past in the collective mind (28). These factors, coupled with widespread misinformation and conspiracy theories (37), which propagated with often little to no reaction from the medical community, medical professional societies, and public health authorities, had a profound impact on the implementation and overall success of the national COVID-19 vaccination campaign in Romania (5).

### Mass media and conspiracy theories

Since the dawn of the COVID-19 epidemic, Romanian media coverage of the subject varied vastly in terms of scientific accuracy. It was generally filled with sensationalist stories (many of which were hypothetical scenarios) mixed with factual news. For the public, this made genuine information difficult to disentangle from alarmist scenarios and sometimes even conspiracy theories (38). Furthermore, many of the anti-vaccine voices that had been present in the public space before the pandemic, were once again offered a platform once the prospect of vaccines became a topic of discussion (39). Their presence was also notable on social media, where conspiracy theories and anti-vaccine views were widely distributed by the members of the public. Furthermore, the feelings of fear and uncertainty that were continuously reinforced on communication channels such as the radio, television, and social media unavoidably impacted the emotional state of the Romanians (40–42), fueling the general mistrust in authorities.

Moreover, the scientific communication efforts on pandemic matters were generally unstructured and inconsistent in Romania (5). No core of highly reputable professionals from within the medical field was assembled in an official capacity as pandemic communicators. Instead, information on pandemic matters was communicated on various media platforms by a wide range of medical and scientific professionals, who varied in terms of reputation, charisma, and even specialized views on topics such as infectious disease management. Furthermore, national media debates on pandemic issues often involved politicians with no formal medical training or scientific expertise on the matter (43).

### Religious communities during the COVID-19 vaccine rollout

The cultural context also played an important role; religious identity and religiosity proved to be important catalysts for public health policy implementation in Romania [for a comprehensive review, see ref. (44)]. Up to 85% of Romanians identify as Orthodox Christians and adhere to the Romanian Orthodox Church, a highly influential institution in the country, which enjoys a high degree of trust from its followers. The restrictions imposed by the Romanian authorities during the pandemic profoundly impacted religious practice. In Romania, the dialogue between governmental authorities and religious organizations was carried out only behind closed doors and with no participation of other stakeholders, such as the press, human rights experts, or representatives of the opposition (45). By comparison, the UK’s strategy aimed at a very transparent public communication, consulting all stakeholders (46) and identifying consensual solutions precisely so that the lifting of restrictions simultaneously met public health requirements, and also those of religious freedom (47).

An illustrative example of the lack of dialogue with religious communities in Romania was the total ban on visits to cemeteries in 2020, from 16 March until 15 May (48). In a highly religious country such as Romania, with most of the Eastern Orthodox believers particularly dedicated to the remembrance of their dead, this measure was acutely felt (44, 48). In addition to the ban on attending places of worship, this was perceived as a direct violation of principles of freedom of religion and belief (44). As a consequence, although most of the Romanian religious leaders have tacitly endorsed the early pandemic restrictions, major divisions were created within the churches, paralleling the broader Romanian society (48). Indeed, this societal polarization extended to all pandemic-related topics, including COVID-19 vaccination.

### Changes in the political landscape

General elections were held in December 2020, at the end of pandemic’s first year, and led to the emergence of a new political party, highly conservative and nationalistic in its ideology: the Alliance for the Union of Romanians (AUR) (49). Within a little over a year of its existence, AUR secured 10% of parliamentary seats, mainly through an electoral rhetoric focused on criticizing the measures for pandemic control, from lockdowns to church closures, and even the mask mandates (50). This party’s campaign heavily capitalized on people’s frustrations and growing distrust in governmental authorities, and on religious feelings stemming from events such as the above-mentioned ban on cemetery visits. After the election, AUR became the main political force opposing the COVID-19 mitigation efforts, with many of their most vocal members actively challenging public health measures and spreading misinformation and disinformation (e.g., the pandemic being fake, the virus being harmless, the COVID-19 death toll being artificially inflated, or even the inexistence of SARS-CoV-2), which on several occasions caused public unrest amongst their supporters (51).

The anti-restriction and anti-vaccine campaign of AUR was also indirectly fueled by the governmental measures that impacted freedom of religion and belief (52). As such, restrictions affecting places of worship, the ban on cemeteries (see above), and the inadequate levels of dialogue between state officials and religious leaders were exploited by right-wing populist voices in the media and further framed by AUR as a threat to the “purity of the nation” (45, 51). Later, once the vaccines became available, the same narrative targeted the COVID-19 vaccination campaign. Moreover, AUR encouraged and further legitimized anti-vaccine voices in Romania (43). Notable examples are the “medical conferences” hosted in the Romanian parliament building which promoted myths and disinformation on vaccines (51, 53).

Altogether, although it is difficult to quantify, the events that occurred during the first year of the pandemic likely shifted public perception towards an incremental dissatisfaction with the way the pandemic was handled by the Romanian authorities, and they further eroded the public trust in COVID-19 control efforts.

## The COVID-19 vaccination campaign: rollout, stakeholder engagement, influence of politics, and public reception

By late 2020, as the prospect of COVID-19 vaccines became reality, Romanian authorities began preparations for the national immunization campaign (29). It was decided that the Romanian military would handle the logistics of the campaign. Historically, the military enjoyed high levels of trust in Romanian society (~70% of citizens at the start of the campaign), therefore their involvement in the vaccination campaign was practical in terms of both organization and public perception (29). With these aspects being covered, other challenges remained [for a detailed description, see ref. (29)], such as prioritizing vaccine recipients, and tackling misinformation and conspiracy theories about vaccines.

To address the challenges of the vaccination campaign and to directly manage the implementation of COVID-19 vaccines in Romania, a National Coordination Committee on COVID-19 Vaccination (CNCAV) was created, reporting directly to the Ministry of Defense and the Prime Minister (54). The committee began the COVID-19 vaccination campaign in Romania on 27 December 2020.

### Early COVID-19 vaccine rollout struggles

In the first months of 2021, the CNCAV faced significant logistical and operational challenges to an optimal vaccine rollout (29). The committee was tasked with coordinating vaccine distribution across the country and needed to rapidly establish efficient infrastructure for vaccine storage, transport, and administration, while navigating a complex landscape of public skepticism, misinformation and disinformation. Public opinion was already unfavorable to a large-scale vaccination campaign (36). Public mistrust in government institutions, stemming from years of political instability and perceived corruption, had eroded confidence in health authorities long before the pandemic (28). This existing skepticism likely served as fertile ground for the rapid spread of misinformation and disinformation regarding vaccines. Conspiracy theories questioning the safety and the actual need of the vaccines proliferated across social media and traditional news platforms, amplifying doubts about their efficacy.

### A promising start, but an abysmal outcome

In the initial stages of the vaccination campaign, Romania achieved one of the fastest COVID-19 vaccine uptakes in the European Union (55). However, the momentum rapidly waned, and vaccine administration plateaued at suboptimal levels, leading to one of the lowest coverage levels in the European Union by the end of 2021 (5). The initial numbers were mainly due to the prioritized vaccination of the healthcare workers and of the vulnerable groups (55). Subsequently, in May 2021, once the quantities of vaccines became sufficient for the total population, the next phase of the campaign began. However, this was the time when the stagnation became evident, mainly driven by public skepticism, which was fueled by misinformation, mistrust in authorities, and inconsistent government messaging (5). By mid-2021, it became clear that vaccine coverage in Romania lagged significantly when compared to other EU countries, with less than 30% of the population being fully vaccinated by autumn 2021 (5).

### Opposition to vaccine and Romanian healthcare professionals

The Romanian healthcare professional community, despite achieving higher vaccination rates, was still confronted with refusals of the vaccine by some health professionals (56). More concerningly, some vocal anti-vaccine doctors played a major role in spreading misinformation, disinformation, and false claims about vaccines (51, 57). These individuals quickly became public figures, gaining visibility through television networks and other media outlets.

Some of these doctors promoted unproven treatments, exaggerated vaccine risks, or downplayed the severity of the pandemic, while others denied the existence of viruses, questioned vaccine efficacy, or spread conspiracy theories (51). Their medical credentials lent credibility to misinformation, thereby misleading the public and fueling vaccine hesitancy. Unfortunately, while the National College of Physicians (the regulatory authority for the medical profession in Romania) has taken steps to address misconduct among physicians, these actions have often been limited and largely ineffective due to the absence of a legal framework that would allow the College to sanction them (57, 58). This underscores the urgent need to revise existing legislation to ensure that professional misconduct, particularly when it poses risks to public health, is addressed promptly and effectively to prevent harmful consequences.

Unlike other EU countries, where misinformation was met with firm sanctions and professional outrage, Romania saw little reaction from the medical professions. Very few voices consistently raised against this flood of misinformation and disinformation (59). Some of these public figures held influential positions in medical organizations or academia, which likely further amplified their reach. While a few individuals faced judicial consequences, most continued to shape public perception with minimal repercussions. Their influence extended beyond healthcare, resonating in religious (see below) and social circles, deepening public distrust in the medical system.

### COVID-19 vaccination and religious communities

The CNCAV’s approach regarding religious organizations aimed at eliciting clear statements in favour of vaccination from their leaders and clergymen (44, 60). This was done through direct messaging in the media, urging the church to take a more active stance in supporting vaccination. However, this approach was complicated by the strained relationship between the government and the church, as the lockdown dramatically impacted religious life during the pandemic and had been imposed without any prior consultation with church leaders (see above) (45). This lack of dialogue during the earlier phases of the pandemic fostered profound mistrust among believers towards government authorities, which extended to the vaccination campaign (44). The Romanian Orthodox Church, is majoritarian (and most influential) in Romania, and one of the best outcomes that public authorities could achieve were endorsements from the Patriarch and other influential religious figures, who urged followers to “listen to the doctors” (61). While this was a positive and reasonable message, it did not explicitly refer to doctors that were in favour of vaccination and not the ones that were contributing to the spread of misinformation (see above).

Efforts by the authorities to involve the Romanian Orthodox Church and other religious groups more actively in the vaccination campaign, such as requesting priests to receive vaccinations live on television or suggesting churches as vaccination sites, did little to sway public opinion (62). These initiatives, while well-intended, failed to resonate with the faithful (44) as the deeply rooted mistrust in government authorities, compounded by the prior lack of consultation with the church during the strict lockdown, overshadowed these gestures (44, 45, 52). The idea of priests being vaccinated on live TV was seen by many as a symbolic action without genuine impact, as it did not address the underlying concerns and skepticism prevalent among the religious population. Similarly, the proposal to use church spaces as vaccination sites did not gain traction, as it was perceived by some as a superficial attempt to align with religious institutions rather than a meaningful engagement with the church’s leadership and community. In the end, these efforts did little to break through the existing mistrust, and the polarization of opinions about vaccination, even among doctors, further diminished their effectiveness (44, 52).

Conspiracy theories, often fueled by online misinformation and religious fundamentalist rhetoric, further deepened mistrust. Notably, on numerous occasions, certain priests [or even bishops, see ref. (44)] discouraged vaccination by suggesting it could lead to physical deformities, such as growing fish scales (63), or other harmful unproven effects (64). The “fish scale sermon” became viral; unfortunately, the news that its unvaccinated author nearly died few months later in the Infectious Diseases Hospital did not (59).

### Politicizing the vaccination campaign

The COVID-19 vaccination campaign in Romania quickly became a highly politicized issue, with statements from the country’s leadership playing a key role in framing the narrative. The Prime Minister and the President both declared in June 2021 that Romania had effectively “ended the pandemic” through vaccination efforts, a claim that was premature and ultimately misleading, as it could be seen few months later by the onset of the fourth Delta wave that followed their declarations and was the most lethal (5). However, such triumphalist messages probably deterred the vaccination efforts, as the public did not see a need to vaccinate since the epidemic ended. On the other hand, opposition parties such as AUR capitalized on this discrepancy by spreading misinformation about the vaccines and using anti-vaccine rhetoric to rally their base (43, 50). Overall, it is likely that the politicization of the vaccination effort not only hindered the campaign’s progress but also deepened societal divisions, allowing misinformation to flourish across political lines and impacting the uptake of COVID-19 vaccines in Romania.

### Inequities in healthcare access and vaccination coverage

The challenges faced by the Romanian COVID-19 vaccination campaign were also compounded by longstanding inequities in access to healthcare that predated the pandemic. Rural communities, low-income groups, and marginalized minorities such as the Roma population often faced barriers of availability and acceptance of care. For example, one study that focused on Central and South-East European countries (including Romania) found that Roma children had substantially lower vaccination coverage compared with non-Roma peers, in some cases less than half for essential vaccines such as DPT, MMR, polio, and BCG (65). This disparity has historically contributed to Romania’s past vulnerability to outbreaks, including the devastating 2016–2019 measles epidemic (28, 66, 67). Overall, the systemic gaps in healthcare access, although difficult to quantify, likely hindered the rollout of COVID-19 vaccines in Romania and the public health efforts in the country.

## The aftermath of the COVID-19 pandemic and its impact on vaccination Romanian public health

By the end of Romania’s COVID-19 vaccination campaign, the country found itself among the least vaccinated nations in the European Union (68). Despite the initial strong start, the vaccination rate plateaued early, and by the end of 2021, only about 40% of the population had received two doses of the vaccine, far behind the EU average of close to 70% (68, 69). Countries such as Portugal and Spain had reached vaccination rates of around 80%, while Romania, along with Bulgaria, remained at the bottom of the EU rankings. The slow uptake left large portions of the population vulnerable to the subsequent waves of the pandemic, contributing to tremendous infection and mortality rates during the Delta variant-driven fourth wave in the autumn of 2021 (70–73).

Post campaign, sentiments towards vaccination in Romania remained highly polarized. While a significant portion of the population saw the benefits of vaccination, a vocal minority continued to claim deep skepticism (51), particularly in rural areas, where access to credible information is limited, and mistrust of government initiatives runs high (74). The deep-rooted vaccine hesitancy that hampered Romania’s COVID-19 vaccination campaign has had long-lasting consequences, one of which was the decline in MMR vaccination coverage which foreshadowed the dramatic 2023 measles outbreak with >21,000 cases and 21 associated deaths by mid-July 2024 (75).

As such, the reluctance to vaccinate, initially tied to COVID-19 vaccination fears, became a broader public health challenge that endangered not only COVID-19 control but also the prevention of other infectious diseases, including measles. The persistence of anti-vaccine narratives, fueled by misinformation spread during the COVID-19 pandemic, left Romania vulnerable to outbreaks, and the public health campaigns to address these issues will likely face substantial challenges in counteract the damage.

## Discussion and recommendations

In December 2022, the Romanian National Committee on COVID-19 Vaccination declared the vaccination campaign a success, branding the report as *“Rovaccination: Mission Accomplished.”* Indeed, the action of the Romanian National Committee on COVID-19 Vaccination can be referred to as a real success in term of organization, communication and overall functioning, the achievements in terms of vaccination coverage cannot. Romania began with a strong initial uptake, but it quickly fell behind other EU countries, ending with one of the lowest vaccination rates in the region (76). Deep-rooted mistrust in government institutions, fueled by years of political instability and amplified by widespread misinformation, likely played a substantial role in the campaign’s stagnation. Efforts to engage key societal stakeholders including religious communities were largely ineffective, with polarized opinions within these influential groups further complicating the campaign.

The politicization of the vaccination effort, along with triumphalist statements from political leaders, created false expectations and undermined public trust when the pandemic continued to devastate the country. This mistrust, combined with inadequate communication strategies and the failure to address anti-vaccine voices within the medical community, left Romania ill-prepared to combat subsequent waves of COVID-19 and other preventable diseases. As a result, the country not only faced a devastating Delta wave (with October 2021 being one of the most lethal months in country’s history), but also a severe measles outbreak in 2024, illustrating the long-term consequences of vaccine hesitancy and the failure to effectively manage public health crises. Romania’s experience highlights the critical need for rebuilding trust in health institutions and addressing the pervasive influence of misinformation to ensure better outcomes in future public health campaigns (77). Indeed, to improve public health outcomes in Romania and elsewhere, several key aspects need to be addressed and planned:

1. *Strengthening trust in public institutions*: Public mistrust in government institutions has been a major barrier to successful health campaigns. Authorities should prioritize transparent communication, engage in consistent dialogue with the public, and involve trusted local leaders, such as religious figures and community influencers, in public health initiatives. This can help rebuild trust and foster a stronger sense of collaboration between citizens and authorities (78).
2. *Combating misinformation*: Misinformation and conspiracy theories have greatly hindered Romania’s vaccination efforts. To counter this, a coordinated national effort is needed to promote science-based information through mainstream and social media channels. Dedicated task forces of medical experts, media professionals, and government representatives should be created and become readily available during major epidemics to monitor, respond to, and debunk false information in real time. Public health campaigns should emphasize the importance of accurate information, using clear and relatable messaging. While this issue is not unique to Romania, tailored strategies will have better outcomes than generalized campaigns which are nonspecific to their audience(s).
3. *Engaging healthcare professionals as partners and advocates*: Healthcare professionals play a crucial role in shaping public opinion on vaccination and other health measures. Providing training and resources for doctors, nurses, and other health workers to effectively communicate the benefits of vaccines and address concerns can improve their role as trusted advocates. Additionally, disciplinary measures should be enforced against medical professionals who spread misinformation to ensure accountability and preserve public trust.
4. *Developing community-centered health campaigns*: Public health efforts should be both national but also local and community-focused. Tailoring campaigns to different regions and demographic groups, especially rural areas where vaccine uptake has been particularly low, can help address specific concerns and cultural factors. Involving local community leaders in the planning and delivery of health interventions is quintessential and can also ensure more personalized and effective outreach.
5. *Enhancing public literacy on preventive health measures*: Improving public understanding of the importance of preventive health activities is essential for long-term health outcomes. Awareness efforts should begin early, including through comprehensive health education in schools, to build a culture of prevention from a young age. Public health messaging must also challenge the misconception that preventive care is only relevant for children, emphasizing that individuals across all age groups benefit from regular engagement with preventive services.
6. *Leveraging technology for health education*: Digital platforms can be powerful tools for health education. Romania should invest in digital literacy programs that teach people to critically evaluate health information online. Additionally, mobile health apps and telemedicine services can be expanded to provide more accessible health information, particularly in rural areas where access to healthcare and trusted information is limited.
7. *Building resilience in the healthcare system*: Romania’s healthcare system has struggled with underfunding and a lack of resources, which has made it vulnerable during public health crises. Increasing investments in healthcare infrastructure, staffing, and resources will be essential to strengthen the system’s capacity to respond to future health challenges. Long-term strategies should focus on improving both preventive care and emergency response mechanisms.
8. *Incorporating religious and cultural sensitivities into public health campaigns*: Given the influential role of religious institutions in Romanian society, public health authorities should work more closely with religious leaders to ensure their involvement in health campaigns. Establishing open channels of dialogue with the Romanian Orthodox Church and other religious groups can foster collaborative health messaging that resonates with believers and mitigates the negative impact of misinformation. Faith-based institutions should be involved from the beginning in any action for pandemic control. Indeed, with the charitable work of the religious communities more often than not involving the betterment of health, such collaborations would be natural continuations which would maximize the public health outcomes of community-centered initiatives.

While Romania’s COVID-19 vaccination campaign faced significant challenges and was ultimately not a success in terms of broad population coverage, the benefits of vaccination for those who accepted it were undeniable. It also proven that the country has all the necessary infrastructure to carry a successful campaign, should the public be convinced by the usefulness of vaccination. By addressing the key aspects above, we believe that significant and measurable improvements can be made, thereby improving the prevention and control of infectious diseases in Romania and enhancing public health in its broader terms. Conversely, failure to address these issues may have devastating spillover effect on vaccination campaigns for diseases that can induce long-term sequelae and mortality in pediatric populations.

It is also important to note the limitations of this current work. As a narrative review based on secondary sources, our analysis depends on the accuracy and objectivity of the materials consulted. Whilst most of the authors were involved in various aspects of pandemic control in Romania (including the COVID-19 vaccination campaign), no primary data collection was conducted, and causal relationships are unable be established with statistical backing. Nonetheless, we believe that the current paper offers a comprehensive account of Romania’s experience that may inform preparedness and response strategies in other countries facing similar challenges. These insights remain valuable even in the absence of primary data, as they highlight critical leverage points for improving public health communication, stakeholder engagement, and vaccine uptake in future crises. Particularly, we identified a deadly triple M association (mistrust, misinformation, and missed opportunities) which, left unresolved or insufficiently addressed, has the potential to derail any national and international prevention campaign. Addressing the critical areas of trust, communication, and community engagement, is the cornerstone of any approach to improve the effectiveness of vaccination efforts globally.
